# Sex and age differences in inflammatory bowel disease patients; a nationwide study based on Iranian Registry of Crohn’s and Colitis (IRCC)

**DOI:** 10.1371/journal.pone.0304792

**Published:** 2024-07-11

**Authors:** Shaghayegh Khanmohammadi, Ali Sheidaei, Sudabeh Alatab, Ozra Tabatabaei-Malazy, Homayoon Vahedi, Fariborz Mansour-Ghanaei, Hafez Fakheri, Farshad Sheikhesmaeili, Anahita Sadeghi, Ali Reza Sima, Amir Anushiravan, Abbas Yazdanbod, Seyed Hamid Moosavy, Iradj Maleki, Hassan Vosooghinia, Masoud Malekzadeh, Reza Malekzadeh

**Affiliations:** 1 Non-Communicable Diseases Research Center, Endocrinology and Metabolism Population Sciences Institute, Tehran University of Medical Sciences, Tehran, Iran; 2 Research Center for Immunodeficiencies, Pediatrics Center of Excellence, Children’s Medical Center, Tehran University of Medical Sciences, Tehran, Iran; 3 Department of Epidemiology and Biostatics, School of Public Health, Tehran University of Medical Sciences, Tehran, Iran; 4 Digestive Disease Research Center, Digestive Disease Research Institute, Tehran University of Medical Sciences, Tehran, Iran; 5 Endocrinology and Metabolism Research Center, Endocrinology and Metabolism Clinical Sciences Institute, Tehran University of Medical Sciences, Tehran, Iran; 6 Gastrointestinal and Liver Diseases Research Center, Guilan University of Medical Sciences, Rasht, Iran; 7 Gut and Liver Research Center, Mazandaran University of Medical Sciences, Sari, Iran; 8 Liver and Digestive Research Center, Kurdistan University of Medical Sciences, Sanandaj, Iran; 9 Sasan Alborz Biomedical Research Center, Masoud Gastroenterology and Hepatology Center, Tehran, Iran; 10 Gastroenterology and Hepatology Department, Digestive Diseases Research Center, Ardabil University of Medical Sciences, Ardabil, Iran; 11 Shahid Mohammadi Hospital, Hormozgan University of Medical Sciences, Bandar Abbas, Iran; 12 Gastroenterology and Hepatology Department, Faculty of Medicine, Ghaem Hospital, Mashhad, Iran; Center for Primary Care and Public Health, SWITZERLAND

## Abstract

**Background:**

Despite the rising prevalence of Inflammatory Bowel Disease (IBD), age and sex differences in its outcomes remain understudied. We investigated age and sex differences in IBD patients using a nationwide study in Iran, the Iranian Registry of Crohn’s and Colitis (IRCC).

**Methods:**

The IRCC is a national registry that gathered information on adult IBD patients since 2017. The collected data included demographic information, medication history, disease activity, comorbidities, diagnosis age, prognosis, the extent of ulcerative colitis (UC), Crohn’s disease (CD) location, and extraintestinal manifestations. The statistical methods included the independent Student’s t-test, Chi-square test, and binary logistic regression, using R version 4.2.2.

**Results:**

Among the 9,392 IBD patients, 7,496 (3,600 females) and 1,896 (808 females) had UC and CD, respectively. Sex difference showed higher odds of active disease in the past six months in male CD patients (OR 1.24 [95%CI 1.03, 1.49]) vs. females, but in male UC patients, the OR was 0.85 [0.78, 0.93]. Severe disease was less likely in CD patients aged 19–59 and >60 vs. <18. Similarly, UC patients <18 had lower odds of severe disease vs. those aged 19–59 and >60.

**Conclusions:**

This study emphasizes the importance of understanding age and sex differences in IBD outcomes. These findings contribute to the ongoing global discussion on IBD management and facilitate the development of targeted interventions and personalized care.

## Introduction

Inflammatory Bowel Disease (IBD) is a chronic relapsing and remitting immune-mediated inflammatory condition and presents a complex and multifaceted landscape of challenges for affected individuals. Crohn’s disease (CD) and ulcerative colitis (UC) are the two main subtypes of IBD [1]. Genetic predisposition, environmental factors, and shifts in lifestyle patterns are associated with IBD occurrence [2]. Based on the results of Global Burden of Disease 2019, in 2019, there were around 4.9 million IBD cases worldwide [3]. Moreover, the age-standardized prevalence of IBD had an increasing trend in more than half of the countries [3]. However, since there is no comprehensive registry system for patients with IBD in most countries, the exact number of people affected by IBD remains unknown. A comparison of population-based data between East and West highlights a rapid increase in the incidence of IBD in the East, while IBD incidence has plateaued in the West.

Moreover, there are notable distinctions in the clinical presentation and course of IBD between these regions, with a higher proportion of patients in the East presenting with complicated disease [4]. For example, CD exhibits a distinct pattern in Asia compared to the West, with a predominant occurrence in men and a notable prevalence of perianal fistulas [5]. Therefore, it is necessary to fully understand the epidemiology, progression, and outcomes of IBD in a diverse population. Recent studies in Iran indicate that the prevalence and incidence of IBD are increasing [6]. A modeling study estimated that subjects suffering from IBD in Iran would shift from 23 thousand cases in 2017 to about 30 thousand cases in 2021, and it will increase to about 69 thousand cases in 2035 [7].

While IBD does not discriminate based on sex or age, the disparities observed in its prevalence, clinical manifestations, and outcomes across different demographic groups, particularly in terms of sex and age, have become increasingly apparent [8]. For instance, the death rate and disability-adjusted life years of IBD are higher in females than in males [3]. Several biological factors, such as sex hormones and sex-dependent (epi)genetic and gut microbiome changes, could explain the sex-related differences in IBD patients. However, biological factors cannot thoroughly explain these differences, and non-biological factors, such as different exposures, access to healthcare, socioeconomic status, and quality of care, are also involved in sex and age differences in IBD patients [8, 9].

While IBD can affect individuals of various ages, specific patterns of age involvement and prevalence are noteworthy. IBD is more commonly diagnosed in younger individuals, and its peak age of onset coincides with the most productive and economically active phase of life [9]. Early-onset disease is associated with more severe forms of IBD. Also, complications of IBD differ among different age groups [9]. For instance, a study in the United States compared the elderly and adult groups. It revealed a lower incidence of perianal disease in the elderly than in the adult group [10].

Despite the significant impact of age and sex differences on IBD patients, these factors have not been studied extensively in Iran in proportion to their importance. Additionally, there is a scarcity of published data on crucial variables like sex and age among IBD patients in our region. Therefore, we aimed to investigate age and sex differences and their effect on the outcomes of IBD patients in Iran by using the Iranian Registry of Crohn’s and Colitis (IRCC). The IRCC registry provided the advantage of quantifying the differences among a large population of IBD patients. The data collected from this registry can be used to address critical questions, such as sex- and age-based differences in the epidemiology, clinical course, comorbidities, and response to current therapies among patients with IBD [11].

## Methods and materials

### Study design

IRCC is a prospective multi-center ongoing registry with the cooperation of more than 400 gastroenterologists from 31 provinces of Iran, which has collected information on adult patients diagnosed with IBD since 2017. The collected data until August 1^st^, 2023, were considered for the current study. The diagnosis of IBD is made according to the 2015 World Gastroenterology guidelines based on clinical imaging, colonoscopy, and pathologic manifestations; a detailed description of this registry was provided previously [11]. This registry collects patient information across five domains as a minimum dataset: patient baseline information, symptoms and quality of life, healthcare service utilization, treatment side effects, and prognosis, in the form of patient reports. Gastroenterologists complete a questionnaire that includes the following information: age of diagnosis, disease subtype, history of IBD-related surgery, extent of UC (according to the Montreal classification system) as the (ulcerative proctitis, left-sided UC, pancolitis) CD location as the (ileum, colon, ileocolon), and extraintestinal manifestations. A research assistant gathers additional information through telephone interviews, which include demographic details, medication history, disease activity, comorbidities, healthcare service utilization, and prognosis. The current study has been performed in accordance with the ethical standards laid down in the Declaration of Helsinki and its later amendments. It has been approved by the Research Ethics Committee affiliated with the Tehran University of Medical Sciences, Tehran, Iran (IR.TUMS.EMRI.REC.1401.152). All persons gave their informed consent before their inclusion in the study. The consent was primarily obtained through a verbal consent process at the first telephonic interview. The process included an explanation of the study’s purpose, potential risks and benefits, participant rights, and an opportunity for participants to ask questions. For participants under 18, explicit consent was obtained from their parents or legal guardians by providing the necessary information (including the nature of the study, its purpose, and any potential risks associated with their child’s participation) to parents or guardians. The authors of the current study did not have access to information that could identify individual participants either during or after the data collection process. S1 Checklist illustrates filled the STROBE checklist.

### Definitions

Study population and cohort characteristics have been described previously [11]. Disease activity during the previous six months was evaluated via the Manitoba IBD Index questionnaire, including a 6-level patient-reported response [12]. The IBD-control-8 questionnaire, a patient-reported outcome measure, assessed patients’ disease activity in the previous two weeks [13]. A score of 14 or less indicated active disease. A treatment information questionnaire was used to determine disease severity. This questionnaire was a comprehensive survey comprising 22 questions regarding four major drug groups: steroids, immunomodulators, 5-ASA, and anti-TNF agents, commonly used by patients with inflammatory bowel disease. The questions were administered via telephonic interviews. However, the interpretation of the answers was considered an outcome, categorized as patient-reported outcomes. Patients who used anti-tumor necrosis factor (anti-TNF) drugs, biologics drugs, or corticosteroids were defined as patients with moderate to severe disease. Comorbidities were extracted from the self-comorbidity questionnaire, inquiring about the history of heart disease, high blood pressure, diabetes, kidney disease, and liver disease. We defined early-onset IBD as a disease diagnosis at ≤18 years of age and elderly-onset as a disease diagnosis at ≥60 years old, based on previous studies [14, 15]. After data extraction, multiple imputation algorithms based on the expectation–maximization (EM) algorithm were employed in cases of missing values.

### Statistical analysis

Continuous variables were reported as mean ± standard deviation (SD) and compared using either independent t-tests or Wilcoxon rank-sum tests. Categorical data were presented as numbers and percentages and compared via Chi-square or Fisher’s exact tests. Following descriptive analysis and initial correlation in univariate form, binary logistic regression was employed for further analyses of binary outcomes. In these comparisons, males were contrasted with females, and patients aged 15–59 years and those over 60 were compared to patients under 18 years old. Statistical analyses and graph creation were conducted using R statistical software version 4.2.2. All tests were two-tailed, and statistical significance was set at p < 0.05.

## Results

**Fig 1:**
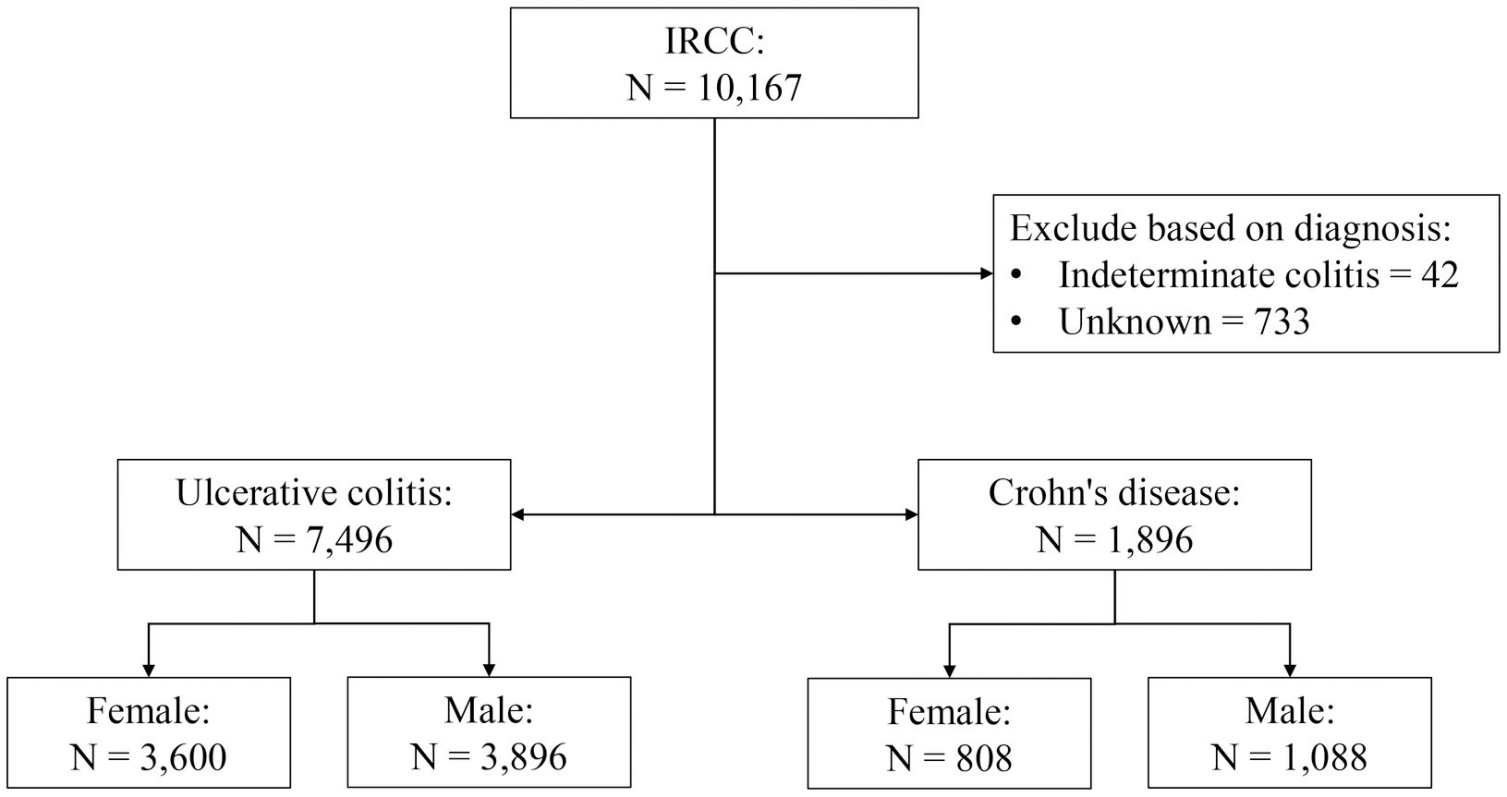
Patients’ selection flowchart.

Out of 10,167 IBD patients enrolled in the IRCC, 42 cases of indeterminate colitis and 733 cases of unknown diagnosis were excluded from the analysis. Among them, 7,496 cases (3,600 females) had UC, and 1,896 cases had CD (808 females) (Fig 1). The baseline characteristics of the patients are summarized in Table 1.

**Table 1:** Basic characteristics of UC and CD patients by sex.

	Female				Male				Total			
Variable	Overall, N = 4,408	UC, N = 3,600	CD, N = 808	p-value	Overall, N = 4,984	UC, N = 3,896	CD, N = 1,88	p-value	Overall, N = 9,392	UC, N = 7,496	CD, N = 1,896	p-value
**Marital status**				**0.009**				**<0.001**				**<0.001**
**Single**	806 (21%)	635 (20%)	171 (24%)		1148 (26%)	834 (24%)	305 (34%)		1904 (24%)	1478 (22%)	476 (29%)	
**Married**	3099 (79%)	2564 (80%)	535 (76%)		3206 (74%)	2602 (76%)	604 (66%)		6305 (76%)	5166 (78%)	1139 (71%)	
**Education**				**0.043**				0.05				**0.002**
**Illiterate**	211 (4.8%)	186 (5.2%)	25 (3.1%)		92 (1.8%)	76 (2.0%)	16 (1.5%)		303 (3.2%)	262 (3.5%)	41 (2.2%)	
**Lower than diploma**	1,003 (23%)	819 (23%)	184 (23%)		874 (18%)	707 (18%)	167 (15%)		1,877 (20%)	1,526 (20%)	351 (19%)	
**Diploma and higher**	3,194 (72%)	2,595 (72%)	599 (74%)		4,018 (81%)	3,113 (80%)	905 (83%)		7,212 (77%)	5,708 (76%)	1,504 (79%)	
**Smoking**				**<0.001**				**<0.001**				**<0.001**
**Never**	4,318 (98%)	3,540 (98%)	778 (96%)		4,025 (81%)	3,174 (81%)	851 (78%)		8,343 (89%)	6,714 (90%)	1,629 (86%)	
**Current smoker**	61 (1.4%)	38 (1.1%)	23 (2.8%)		537 (11%)	384 (9.9%)	153 (14%)		598 (6.4%)	422 (5.6%)	176 (9.3%)	
**Former smoker**	29 (0.7%)	22 (0.6%)	7 (0.9%)		422 (8.5%)	338 (8.7%)	84 (7.7%)		451 (4.8%)	360 (4.8%)	91 (4.8%)	
**Opium use**				0.075				**<0.001**				**<0.001**
**Never**	4,391 (100%)	3,589 (100%)	802 (99%)		4,829 (97%)	3,796 (97%)	1,033 (95%)		9,220 (98%)	7,385 (99%)	1,835 (97%)	
**Current user**	13 (0.3%)	9 (0.3%)	4 (0.5%)		94 (1.9%)	51 (1.3%)	43 (4.0%)		107 (1.1%)	60 (0.8%)	47 (2.5%)	
**Former user**	4 (<0.1%)	2 (<0.1%)	2 (0.2%)		61 (1.2%)	49 (1.3%)	12 (1.1%)		65 (0.7%)	51 (0.7%)	14 (0.7%)	
**Appendectomy**	276 (6.3%)	169 (4.7%)	107 (13%)	**<0.001**	371 (7.4%)	221 (5.7%)	150 (14%)	**<0.001**	647 (6.9%)	390 (5.2%)	257 (14%)	**<0.001**
**Age at diagnosis, yr, (mean±SD)**	32.51 (12.74)	32.62 (12.53)	31.97 (13.68)	0.2	32.81 (133.30)	33.20 (13.12)	31.40 (13.86)	**<0.001**	32.67 (13.04)	32.92 (12.84)	31.64 (13.78)	**<0.001**
**Age at diagnosis**				**<0.001**				**<0.001**				**<0.001**
**< = 18**	442 (10%)	322 (9.0%)	120 (15%)		519 (10%)	344 (8.9%)	175 (16%)		961 (10%)	666 (8.9%)	295 (16%)	
**19–39**	2,849 (65%)	2,393 (67%)	456 (58%)		3,163 (64%)	2,530 (65%)	633 (60%)		6,012 (64%)	4,923 (66%)	1,089 (59%)	
**40–59**	916 (21%)	740 (21%)	176 (23%)		1,023 (21%)	820 (21%)	203 (19%)		1,939 (21%)	1,560 (21%)	379 (21%)	
**60+**	168 (3.8%)	138 (3.8%)	30 (3.8%)		241 (4.9%)	190 (4.9%)	51 (4.8%)		409 (4.4%)	328 (4.4%)	81 (4.4%)	
**UC Extension**												
**Proctitis**	-	390 (19%)	-		-	422 (19%)	-		-	812 (19%)	-	
**Left sided colitis**	-	759 (36%)	-		-	713 (32%)	-		-	1,472 (34%)	-	
**Pancolitis**	-	946 (45%)	-		-	1,105 (49%)	-		-	2,051 (47%)	-	
**Crohn’s Location**												
**Ileal**	-	-	162 (34%)		-	-	219 (36%)		-	-	381 (35%)	
**Colonic**	-	-	114 (24%)		-	-	106 (17%)		-	-	220 (20%)	
**Ileocolonic**	-	-	197 (42%)		-	-	283 (47%)		-	-	480 (44%)	
**Corticosteroids**				**<0.001**				**<0.001**				**<0.001**
**Never**	2,293 (53%)	1,945 (55%)	348 (44%)		2,848 (58%)	2,303 (60%)	545 (51%)		5,141 (56%)	4,248 (58%)	893 (48%)	
**Current use**	473 (11%)	364 (10%)	109 (14%)		515 (11%)	393 (10%)	122 (11%)		988 (11%)	757 (10%)	231 (12%)	
**Previous use**	1,539 (36%)	1,204 (34%)	335 (42%)		1,525 (31%)	1,129 (30%)	396 (37%)		3,064 (33%)	2,333 (32%)	731 (39%)	
**Anti-TNF agents’ consumption**				**<0.001**				**<0.001**				**<0.001**
**Never**	3,512 (81%)	3,020 (85%)	492 (61%)		3,891 (79%)	3,249 (84%)	642 (60%)		7,403 (80%)	6,269 (85%)	1,134 (60%)	
**Current use**	651 (15%)	387 (11%)	264 (33%)		788 (16%)	418 (11%)	370 (34%)		1,439 (15%)	805 (11%)	634 (34%)	
**Previous use**	192 (4.4%)	146 (4.1%)	46 (5.7%)		253 (5.1%)	189 (4.9%)	64 (5.9%)		445 (4.8%)	335 (4.5%)	110 (5.9%)	
**IBD- related surgery**	207 (20%)	89 (12%)	118 (41%)	**<0.001**	295 (24%)	116 (14%)	179 (42%)	**<0.001**	502 (22%)	205 (13%)	297 (42%)	**<0.001**
**Extraintestinal manifestation**	2,426 (55%)	2,043 (57%)	383 (47%)	**<0.001**	2,618 (53%)	2,108 (54%)	510 (47%)	**<0.001**	5,044 (54%)	4,151 (55%)	893 (47%)	**<0.001**
**PSC**	115 (2.6%)	104 (2.9%)	11 (1.4%)	**0.014**	167 (3.4%)	153 (3.9%)	14 (1.3%)	**<0.001**	282 (3.0%)	257 (3.4%)	25 (1.3%)	**<0.001**
**Comorbidities**												
**Heart disease**	194 (4.4%)	143 (4.0%)	51 (6.3%)	0.003	242 (4.9%)	186 (4.8%)	56 (5.1%)	0.6	436 (4.6%)	329 (4.4%)	107 (5.6%)	0.02
**Hypertension**	390 (8.8%)	320 (8.9%)	70 (8.7%)	0.8	331 (6.6%)	265 (6.8%)	66 (6.1%)	0.4	721 (7.7%)	585 (7.8%)	136 (7.2%)	0.4
**Diabetes**	202 (4.6%)	167 (4.6%)	35 (4.3%)	0.7	172 (3.5%)	134 (3.4%)	38 (3.5%)	>0.9	374 (4.0%)	301 (4.0%)	73 (3.9%)	0.7
**Kidney disease**	148 (3.4%)	114 (3.2%)	34 (4.2%)	0.14	238 (4.8%)	185 (4.7%)	53 (4.9%)	0.9	386 (4.1%)	299 (4.0%)	87 (4.6%)	0.2
**Liver disease**	303 (6.9%)	264 (7.3%)	39 (4.8%)	**0.011**	414 (8.3%)	352 (9.0%)	62 (5.7%)	**<0.001**	717 (7.6%)	616 (8.2%)	101 (5.3%)	**<0.001**

n (%); Mean (SD)

Pearson’s Chi-squared test; Fisher’s exact test; Welch Two Sample t-test

Pearson’s Chi-squared test; Welch Two Sample t-test; Fisher’s exact test

PSC: Primary sclerosing cholangitis

### Active disease during the past two weeks

Our analysis showed no statistical difference in active disease during the past two weeks between males and females and among different age groups in CD patients (Tables 1 and 2).

**Table 2:** Basic characteristics of UC and CD patients by age.

	< = 18				19–59				60+			
Variable	Overall, N = 961	UC, N = 666	CD, N = 295	p-value	Overall, N = 7,951	UC, N = 6,483	CD, N = 1,468	p-value	Overall, N = 409	UC, N = 328	CD, N = 81	p-value
**Sex**				**0.028**				**<0.001**				0.4
**Female**	442 (46%)	322 (48%)	120 (41%)		3,765 (47%)	3,133 (48%)	632 (43%)		168 (41%)	138 (42%)	30 (37%)	
**Male**	519 (54%)	344 (52%)	175 (59%)		4,186 (53%)	3,350 (52%)	836 (57%)		241 (59%)	190 (58%)	51 (63%)	
**Marital status**				**0.002**				**0.054**				**0.2**
**Single**	570 (69%)	378 (66%)	192 (76%)		1347 (19%)	1083 (19%)	264 (21%)		17 (5%)	12 (4%)	5 (7%)	
**Married**	255 (31%)	196 (34%)	59 (24%)		5650 (81%)	4669 (81%)	981 (79%)		349 (95%)	287 (96%)	62 (93%)	
**Education**				0.3				**0.018**				0.066
**Illiterate**	11 (1.1%)	10 (1.5%)	1 (0.3%)		195 (2.5%)	169 (2.6%)	26 (1.8%)		96 (23%)	83 (25%)	13 (16%)	
**Lower than diploma**	214 (22%)	146 (22%)	68 (23%)		1,504 (19%)	1,253 (19%)	251 (17%)		149 (36%)	122 (37%)	27 (33%)	
**Diploma and higher**	736 (77%)	510 (77%)	226 (77%)		6,252 (79%)	5,061 (78%)	1,191 (81%)		164 (40%)	123 (38%)	41 (51%)	
**Smoking**				0.6				**<0.001**				**0.002**
**Never**	894 (93%)	621 (93%)	273 (93%)		7,100 (89%)	5,838 (90%)	1,262 (86%)		288 (70%)	239 (73%)	49 (60%)	
**Current smoker**	53 (5.5%)	37 (5.6%)	16 (5.4%)		502 (6.3%)	362 (5.6%)	140 (9.5%)		36 (8.8%)	21 (6.4%)	15 (19%)	
**Former smoker**	14 (1.5%)	8 (1.2%)	6 (2.0%)		349 (4.4%)	283 (4.4%)	66 (4.5%)		85 (21%)	68 (21%)	17 (21%)	
**Opium use**				>0.9				**<0.001**				**<0.001**
**Never**	960 (100%)	665 (100%)	295 (100%)		7,816 (98%)	6,387 (99%)	1,429 (97%)		375 (92%)	314 (96%)	61 (75%)	
**Current user**	1 (0.1%)	1 (0.2%)	0 (0%)		78 (1.0%)	48 (0.7%)	30 (2.0%)		27 (6.6%)	11 (3.4%)	16 (20%)	
**Former user**	0 (0%)	0 (0%)	0 (0%)		57 (0.7%)	48 (0.7%)	9 (0.6%)		7 (1.7%)	3 (0.9%)	4 (4.9%)	
**Appendectomy**	58 (6.0%)	22 (3.3%)	36 (12%)	**<0.001**	549 (6.9%)	352 (5.4%)	197 (13%)	**<0.001**	30 (7.3%)	14 (4.3%)	16 (20%)	**<0.001**
**UC Extension**												
**Proctitis**	-	54 (14%)	-		-	720 (19%)	-		-	37 (17%)	-	
**Left sided colitis**	-	111 (29%)	-		-	1,264 (34%)	-		-	97 (45%)	-	
**Pancolitis**	-	221 (57%)	-		-	1,748 (47%)	-		-	80 (37%)	-	
**Crohn’s Location**												>0.9
**Ileal**	56 (34%)	-	56 (34%)		-	-	289 (34%)		-	-	26 (47%)	
**Colonic**	23 (14%)	-	23 (14%)		-	-	178 (21%)		-	-	14 (25%)	
**Ileocolonic**	84 (52%)	-	84 (52%)		-	-	376 (45%)		-	-	15 (27%)	
**Corticosteroids**				**0.009**				**<0.001**				0.4
**Never**	445 (47%)	330 (50%)	115 (40%)		4,387 (56%)	3,689 (58%)	698 (48%)		286 (75%)	225 (74%)	61 (81%)	
**Current use**	138 (15%)	92 (14%)	46 (16%)		813 (10%)	635 (10.0%)	178 (12%)		33 (8.7%)	29 (9.5%)	4 (5.3%)	
**Previous use**	362 (38%)	233 (36%)	129 (44%)		2,600 (33%)	2,035 (32%)	565 (39%)		61 (16%)	51 (17%)	10 (13%)	
**Anti-TNF agents’ consumption**				**<0.001**				**<0.001**				**0.001**
**Never**	661 (70%)	516 (79%)	145 (50%)		6,371 (81%)	5,464 (85%)	907 (62%)		334 (85%)	279 (89%)	55 (71%)	
**Current use**	242 (26%)	108 (16%)	134 (46%)		1,129 (14%)	665 (10%)	464 (32%)		46 (12%)	29 (9.2%)	17 (22%)	
**Previous use**	45 (4.7%)	32 (4.9%)	13 (4.5%)		378 (4.8%)	291 (4.5%)	87 (6.0%)		12 (3.1%)	7 (2.2%)	5 (6.5%)	
**IBD- related surgery**	71 (32%)	22 (18%)	49 (49%)	**<0.001**	411 (21%)	174 (13%)	237 (44%)	**<0.001**	20 (27%)	9 (18%)	11 (48%)	**0.011**
**having extraintestinal manifestation**	513 (53%)	362 (54%)	151 (51%)	0.4	411 (21%)	174 (13%)	237 (44%)	**<0.001**	269 (66%)	219 (67%)	50 (62%)	0.4
**PSC**	43 (4.5%)	42 (6.3%)	1 (0.3%)	**<0.001**	228 (2.9%)	206 (3.2%)	22 (1.5%)	**<0.001**	11 (2.7%)	9 (2.7%)	2 (2.5%)	>0.9
**Comorbidities**												
**Heart disease**	18 (1.9%)	11 (1.7%)	7 (2.4%)	0.4	307 (3.9%)	237 (3.7%)	70 (4.8%)	**0.046**	110 (27%)	81 (25%)	29 (36%)	**0.043**
**Hypertension**	24 (2.5%)	15 (2.3%)	9 (3.1%)	0.5	550 (6.9%)	446 (6.9%)	104 (7.1%)	0.8	143 (35%)	124 (38%)	19 (23%)	**0.015**
**Diabetes**	8 (0.8%)	8 (1.2%)	0 (0%)	0.12	309 (3.9%)	252 (3.9%)	57 (3.9%)	>0.9	55 (13%)	40 (12%)	15 (19%)	0.14
**Chronic renal disease**	27 (2.8%)	16 (2.4%)	11 (3.7%)	0.3	323 (4.1%)	253 (3.9%)	70 (4.8%)	0.13	31 (7.6%)	28 (8.5%)	3 (3.7%)	0.14
**Liver disease**	80 (8.3%)	73 (11%)	7 (2.4%)	**<0.001**	609 (7.7%)	525 (8.1%)	84 (5.7%)	**0.002**	22 (5.4%)	16 (4.9%)	6 (7.4%)	0.4

n (%); Mean (SD)

Pearson’s Chi-squared test; Fisher’s exact test; Welch Two Sample t-test

Pearson’s Chi-squared test; Welch Two Sample t-test; Fisher’s exact test

PSC: Primary sclerosing cholangitis

**Fig 2:**
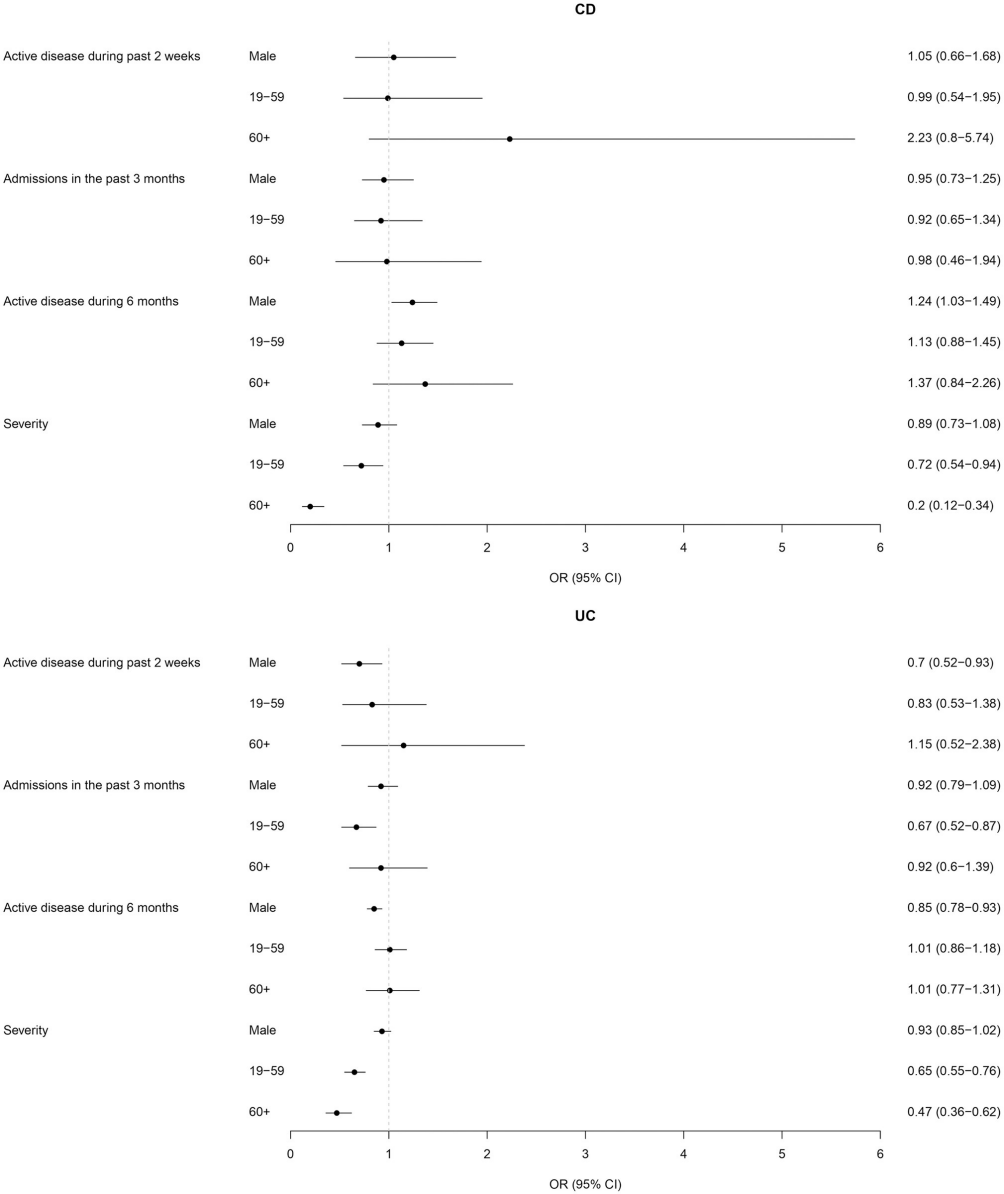
The odds ratios for the association of age and sex with disease activity, severity, and hospitalization.

Among UC patients, males were less likely to have active disease during the past two weeks, with an odds ratio (OR) of 0.7 and a 95% Confidence interval (95%CI) between 0.52 and 0.93 compared to females. However, similar to what was observed in CD patients, no statistical difference in active disease during the past two weeks was seen among age groups in UC patients (Fig 2).

### Hospital admissions during the past three months

Our analysis revealed no statistically significant differences in this variable among different age groups or sexes in patients with CD. Male UC patients showed no statistical difference in hospital admission in the past three months compared to female UC patients. The odds of hospital admission during the past three months were 0.67 times lower (95%CI 0.52, 0.87) in UC patients between 19 and 59 years than in patients under 18. However, this association was not seen in patients older than 60 years compared to patients under 18 years (Fig 2).

### Active disease during the past six months

After logistic regression analysis, our results showed that the odds of active disease among CD patients during the past six months were higher in males than females [OR 1.24, (95% CI 1.03, 1.49)]. No statistical difference in active disease during the past six months was seen among age groups in CD patients. Among UC patients, males were less likely to have active disease during the past six months [OR 0.85, (95%CI 0.78,0.93)] compared to females. Similar to CD patients, no statistical difference in active disease during the past six months was seen among age groups in UC patients (Fig 2).

### Disease severity

We found that the difference in disease severity between male and female groups in CD patients was not statistically significant. However, compared to CD patients under the age of 18 years, patients between 19 and 59 years [OR 0.72, (95%CI 0.54,0.94)] and older [OR 0.2, (95%CI 0.12,0.34)] had lower odds for disease severity. Similar to CD patients, no statistical difference was found between male and female groups in terms of disease severity, and the odds of severe disease were lower in UC patients under the age of 18 years than in patients between 19 and 59 years [(OR 0.65, (95%CI 0.55,0.76)] and ≥ 60 years [OR 0.47, (95%CI 0.36, 0.62)] (Fig 2).

### Comorbidities

**Fig 3:**
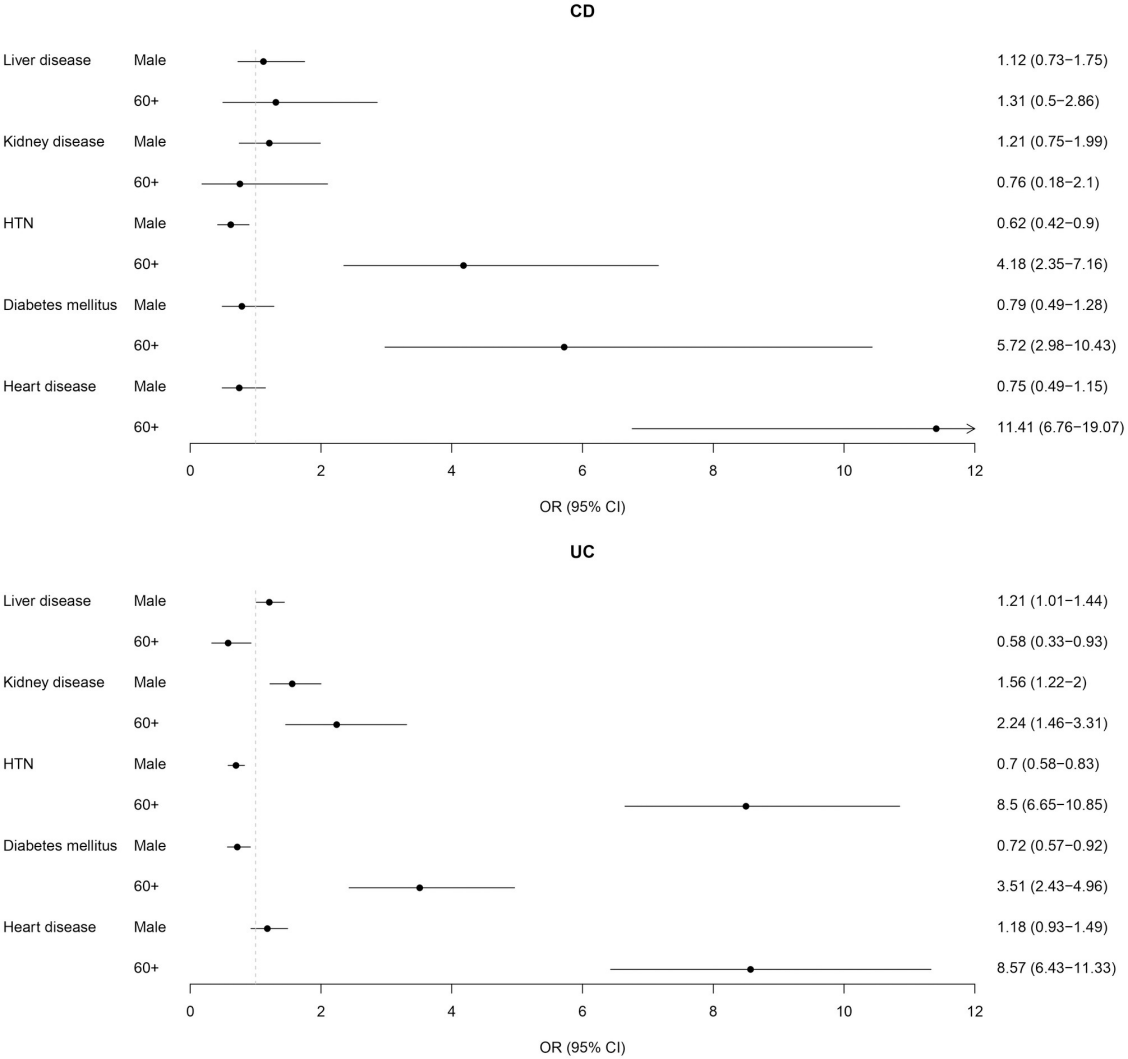
The odds ratios for the association of age and sex with comorbidities.

The study found significant associations between various comorbidities and age groups among patients with CD and UC. Older patients (above 60 years) were more likely to have heart diseases, hypertension, diabetes mellitus, and kidney diseases compared to those under 18 years. Additionally, females generally exhibited higher odds of hypertension in both CD and UC patients and higher odds of diabetes mellitus in UC patients. However, there were no significant differences in the likelihood of heart diseases between males and females in both CD and UC patients, and also in the likelihood of. Kidney and liver diseases between genders in CD patients, while male UC patients had higher odds of kidney and liver diseases. Moreover, liver diseases were less likely to occur in UC patients over 60 years old compared to those under 18 years (Fig 3).

## Discussion

In this study, we evaluated age and sex differences in Iranian patients with IBD and their effects on disease outcomes using IRCC data. We found that UC was more prevalent than CD in our population; more males were diagnosed with CD, but it seems that UC affected females and males approximately equally. Also, the odds of active disease in male CD patients during the past six months were higher than in females. In contrast, female patients with UC had a higher chance of active disease during the past two weeks and six months than male patients. After comparing different age groups within the registry, we found that the odds of disease severity were lower in UC and CD patients between 19 and 59 years and older than in patients under 18. Also, patients between 19 and 59 years were at lower risk for hospital admissions during the past three months than patients under 18.

Similar to previous studies in Iran, Asia, and the Middle East region [16], in our registry, UC was more prevalent than CD [7496 UC patients vs. 1896 CD patients]; also, the age at diagnosis for UC and CD was similar. Our study’s age at diagnosis for UC and CD was 32.92 (SD: 12.84) and 31.64 (SD: 13.78), respectively. Previous studies have shown that the age of onset was 33.6 for UC and 32.3 for CD in Iran [16]. In our study, UC seems to have a female dominance pattern in Iran [16, 17], which was similar to others, while in contrast to our study, CD shows a male dominance pattern [16].

Sex-specific variations in IBD have been documented across disease presentation, progression, complications, medical and surgical treatment approaches, adherence levels, and psychosocial functioning [18]. Although substantial evidence supports certain aspects, conflicting data exist for other dimensions of the condition [18]. For instance, two multi-center observational cohorts combined showed that early-onset CD exhibited a higher incidence among males [19]. Moreover, mortality due to colorectal cancers was more frequent in male IBD patients compared to female IBD patients [18]. In our results, in CD patients, males were more likely to have active disease during the past six months than females, while in UC patients, females had higher odds for active disease during the past two weeks and six months. The potential role of sex as a sole risk factor for increased disease activity and a more complex disease course has received limited attention in existing studies and had conflicting results. In a population-based study conducted by Romberg-Camps et al., involving the examination of data sets from 1,187 patients, no evidence supporting sex as a prognostic factor for severe disease progression was found.

Conversely, a longitudinal study analyzed data from 269 CD patients over 10 years and identified male sex as an apparent risk factor for a complicated disease course [OR, 2.6, (95%CI 1.17, 5.75)] [20]. However, an examination of remission rates in relation to sex within a large German cohort of IBD patients revealed significantly higher remission rates in male UC patients compared to their female counterparts [21]. Similarly, a study involving a German cohort of 1,032 CD patients arrived at a parallel conclusion, indicating that women with CD exhibited increased disease activity compared to men [22].

Previous studies have shown that pediatric patients frequently present with more extensive disease involvement, whereas older adults may experience a milder and more indolent course. Also, the therapeutic approach varies significantly based on the age at onset, with a notable preference for earlier and more frequent utilization of immunosuppressives and, to a certain extent, biologicals, compared to patients with onset in later years, particularly evident in individuals with CD. In our findings, younger patients tended to exhibit a more aggressive disease course, characterized by heightened disease activity compared to older patients. These differences could be attributed to age-related variations in immune response, the composition of gut microbiota, and genetic predispositions [9, 23].

It has been shown that extraintestinal manifestations (affecting many patients) [24], such as arthritis, skin issues, ocular manifestations, arthropathy, and osteopenia, were more prevalent in females. Males were more likely to experience ileal disease and require ileocolic resection more frequently [19]. Moreover, males are at higher risk for spondylitis, primary sclerosing cholangitis (PSC), and secondary amyloidosis than females [25, 26]. A cohort study conducted in the United States revealed that being female was identified as a risk factor for non-fistulizing perianal CD [27]. In our study, PSC was more seen in male patients with UC than in females; however, both sexes with CD showed approximately the same prevalence of PSC. These differences could be explained by differences in environmental factors and genetic predispositions in different populations.

Several studies examining the impact of sex on patient responses to biologics in IBD have produced varying results. A systematic review of 39 studies focusing on adalimumab revealed a higher likelihood of loss of response in males, necessitating dose escalation [28]. Conversely, a retrospective cohort analysis of 210 patients with Crohn’s disease treated with infliximab found that males had a 66% reduced risk of clinical response failure [29]. Another study with a smaller cohort of 47 ulcerative colitis patients treated with infliximab reported no sex-based differences in response [30]. However, a separate investigation involving steroid-refractory UC patients demonstrated that females, in comparison to males, had a 3.5-fold higher likelihood of achieving long-term remission with infliximab [31].

To the best of our knowledge, this is the first study evaluating age and sex differences in IBD patients in Iran within IRCC. Demographic and regional diversity, large sample size, validated and reliable questionnaires based on accepted international minimum datasets, and reflection of real-world clinical settings are among the strengths of our study. However, our study had several limitations. Missing data, confounding variables, and lack of access to detailed medical records of patients both currently and before establishing a national electronic medical record system are among the limitations of our study.

## Conclusion

In conclusion, our comprehensive analysis of sex and age differences in IBD patients sheds light on multifaceted differences in disease activity, disease severity, and hospital admission rates between males and females and among different age groups. Recognizing these variations is pivotal for tailoring interventions to meet the unique needs of each sex and age group, ultimately improving patient outcomes and quality of life. Future research endeavors should continue to unravel the intricate interplay of biological and sociocultural factors contributing to age- and sex-specific differences in IBD, providing a foundation for more targeted and personalized approaches to patient care.

## Supporting information

S1 Checklist Filled the STROBE checklist. (PDF)
